# Membranes in Lithium Ion Batteries

**DOI:** 10.3390/membranes2030367

**Published:** 2012-07-04

**Authors:** Min Yang, Junbo Hou

**Affiliations:** Institute for Critical Technology and Applied Science, Virginia Tech, Blacksburg, VA 24061, USA; Email: min.yang68@yahoo.com

**Keywords:** lithium ion battery, Li ion conductor, separator, ceramic, polymer

## Abstract

Lithium ion batteries have proven themselves the main choice of power sources for portable electronics. Besides consumer electronics, lithium ion batteries are also growing in popularity for military, electric vehicle, and aerospace applications. The present review attempts to summarize the knowledge about some selected membranes in lithium ion batteries. Based on the type of electrolyte used, literature concerning ceramic-glass and polymer solid ion conductors, microporous filter type separators and polymer gel based membranes is reviewed.

## 1. Introduction

**Figure 1:**
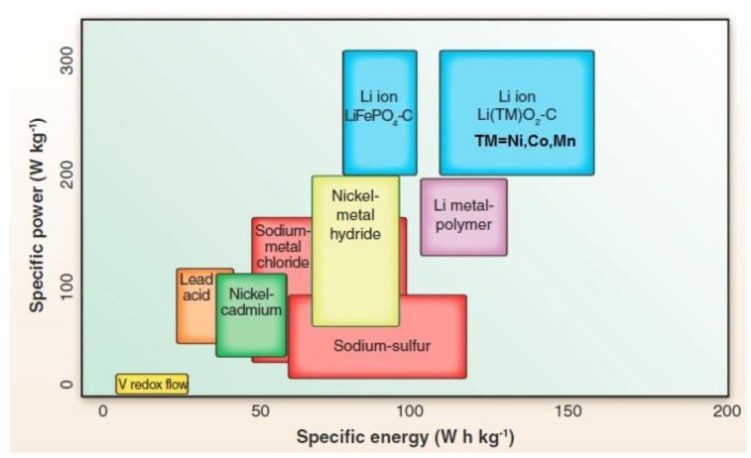
Ragone plots (power *vs.* energy density) for different rechargeable batteries [3].

Since the first primary lithium ion batteries (LIBs) became commercially available in 1991, LIBs caught on quickly and have become the main power sources on the consumer electronics market [1,2]. LIBs are characterized by high specific energy and high specific power (Figure 1), which are the advantages that most other electrochemical energy storage technologies cannot offer [3]. In addition, some other advantages such as high efficiency, long life cycle and low self-discharge rate make lithium-ion batteries well suited for applications such as energy storage grid and electric transportation. Despite the overall advantages, scaling up LIB technology for these applications is still problematic due to safety, costs, operational temperature and materials availability [4]. 

**Figure 2:**
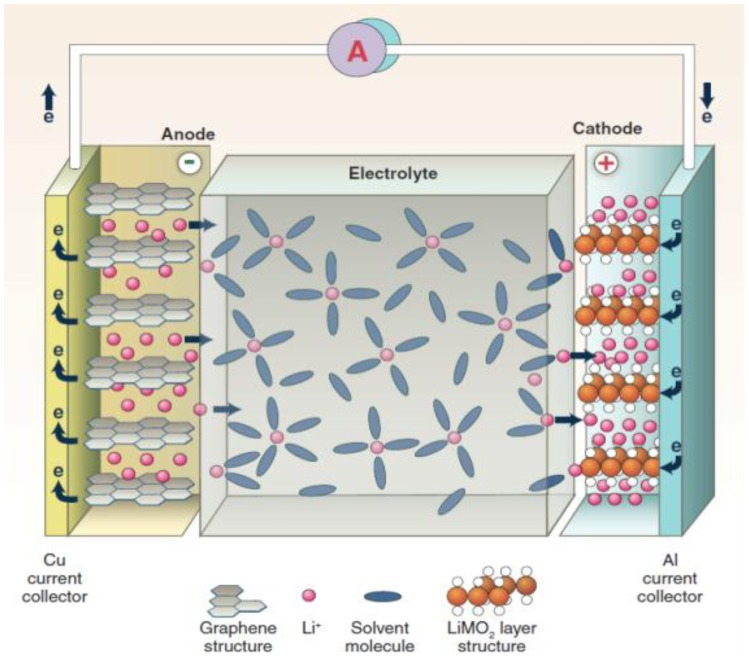
Schematic of a lithium ion battery (LIB) consisting of the negative electrode (graphitic carbon) and positive electrode (Li-intercalation compound) [5].

Figure 2 schematically shows a typical LIB [5], which consists of a cathode (LiMn_1.5_Ni_0.5_O_4_ spinel; layered structure LiMO_2_; LiMPO_4_ olivines, M = Mn, Fe and Co) and an anode (intercalated graphite; alloying materials Si and Sn), together with the electrolyte that allows Li ion transport but prevents electrodes from electronic contact [6]. During charging, Li deintercalates from the cathode and inserts into the anode. When discharging, Li intercalates into the cathode. In the processes of charge/discharge, Li ions transport between the anode and the cathode, which allows electrochemical energy storage within the battery and the conversion of chemical energy into electrical energy. 

The electrolyte usually functions as an electronic separator and ionic conductor between cathode and anode. It may consist of solvent, salt, separator, additive, and/or a solid ion-conducting membrane or a combination thereof. As in other electrochemical devices, the electrolyte should be durable in highly reductive and oxidative environments, highly ionic-conductive, and facilitate electrochemical reactions. Accordingly, if a liquid electrolyte, at least including solvent and salt, is used, an additional membrane is required to electronically separate two electrodes. At the same time, this membrane must be porous and allow the liquid electrolyte to flow through. In a commercial LIB, a porous plastic film as separator is soaked in LiPF_6_ which is dissolved in a mixture of organic solvents such as ethylene carbonate (EC), ethyl methyl carbonate (EMC), or diethyl carbonate (DEC). If the membrane itself is a Li ion conductor, the liquid electrolyte is not a necessity. Another case is to incorporate the liquid electrolyte into the polymer matrix to form a polymer gel electrolyte. These are the most common types of membranes used in a LIB. The main function of these membranes is to prevent the positive and negative electrodes electrically contacting each other, and allow rapid ionic transport to complete the circuit for the passage of current in lithium ion batteries. Therefore, they play very important roles in lithium ion batteries, and may affect the electrochemical energy efficiency. 

## 2. Solid Li Ion Conductors

To simplify the cell design, and improve safety and durability, a solid electrolyte was used to eliminate the need of the liquid electrolyte. Two general classes of materials used for solid electrolytes in lithium-ion batteries include inorganic ceramics and organic polymers. The most obvious difference between these classes is the mechanical properties. Polymers are generally easier to process than ceramics, which reduce the fabrication costs. On the other hand, ceramics are more suitable for high temperature or other aggressive environments.

### 2.1. Ceramic-Glass

#### 2.1.1. Na Super-ionic Conductor (NASICON) Structure

**Figure 3:**
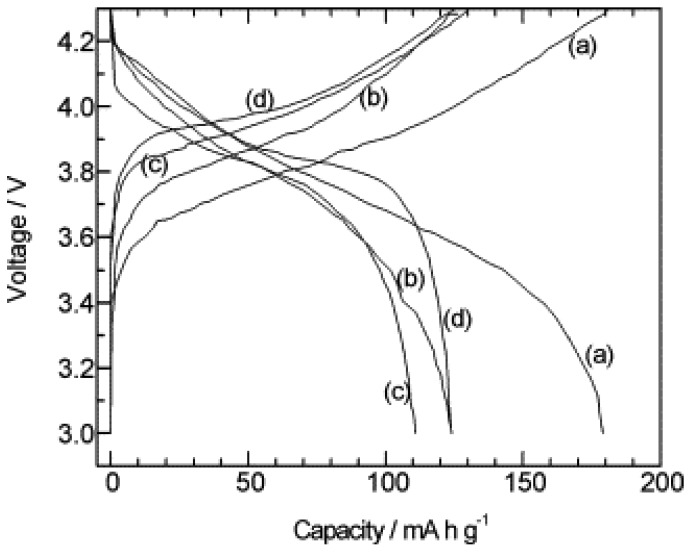
Charge and discharge curves of Li/PEO/LTAP/LiCoO_2_ cells at 50 °C at different annealing temperatures: (**a**) as-deposited; (**b**) 300 °C; (**c**) 400 °C and (**d**) 500 °C [15].

Among the LiM_2_(PO_4_)_3_ (M = Ti, Zr, Ge, Hf) NASICONs, the Ti-compound is supposed to exhibit high lithium ion conductivity at room temperature, due to its lower volume resistivity [7], which means the lowest cell volume of LiTi_2_(PO_4_)_3_ should show highest conductivity. Because the NASICON structure permits a wide range of ion substitution at the Ti and P sites making this structure a versatile family of solids, a lot of work focusing on substitution has been performed attempting to improve Li ion conductivity [8,9,10,11,12]. So far, the highest conductivity based on NASICON structure has been observed for Li_1+x_Al_x_Ge_2−x_(PO_4_)_3_ (LAGP), which can reach 10^−4 ^S cm^−1^ [9]. Another commonly used phosphate electrolyte is Li_1+x_Ti_2−x_Al_x_(PO_4_)_3_ (LTAP) [11,13], and its modification at P sites (*i.e.*, Li_1+x+y_Ti_2−x_Al_x_Si_y_(PO_4_)_3−y_) [14]. Figure 3 shows the application of LTAP as the electrolyte in lithium-ion batteries [15].

#### 2.1.2. Garnet Structure

The nominal chemical compositions for this kind of structure are Li_5_La_3_M_2_O_12_ (M = Nb, Ta) and Li_6_ALa_2_M_2_O_12_ (A = Ca, Sr, Ba; M = Nb, Ta). Usually the Li ion conductivity based on the garnet structure is within 10^−7^ to 10^−5^ S cm^−1^ at room temperature [16,17,18]. The highest conductivity about 10^−4^ S cm^−1^ at room temperature has been obtained from Li_5_La_3_Ta_2_O_12 _with La sites substituted by Ba and/or Sr [19]. The unique feature of garnet-type materials may be that the total and bulk conductivities are nearly identical, which implies the grain boundary resistance should be very small. For example, Li_6_SrLa_2_Ta_2_O_12_ and Li_6_BaLa_2_Ta_2_O_12_ exhibit mainly bulk ionic conductivities of 8.9 × 10^−6^ and 5.4 × 10^−5^ S cm^−1^ at 22 °C, respectively [20]. According to bond valence models, the Li^+^ ion transport pathways in Li_5_La_3_M_2_O_12_ is directly related to the fully occupied octahedral sites [21], which indicates a vacancy-type ion transport is expected to be the dominant contribution. If this is the case, one should be careful when such membrane is used as the electrolyte in LIBs. 

#### 2.1.3. Perovskite Structure

**Figure 4:**
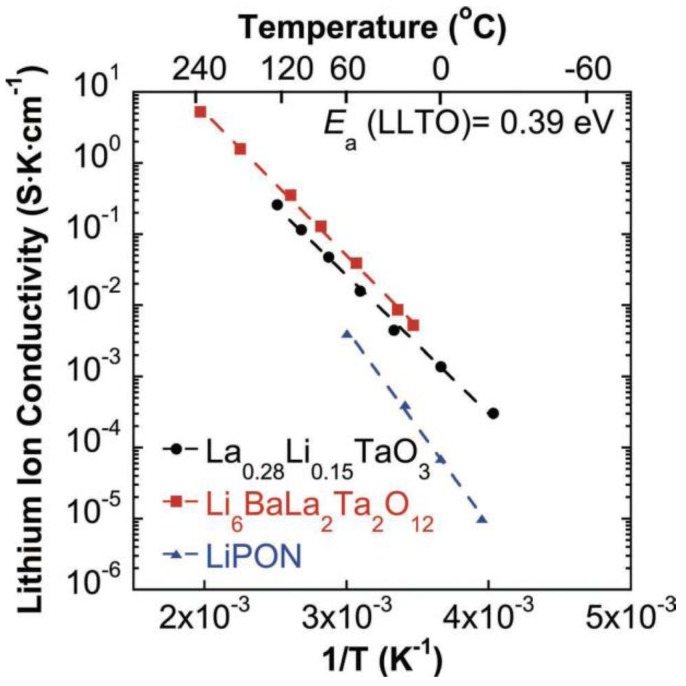
Arrhenius plot for the lithium-ion conductivity of La_0.281_Li_0.155_TaO_3_ compared with data for Li_6_BaLa_2_Ta_2_O_12_ and lithium phosphorus oxy-nitride (LiPON) [27].

The perovskite (ABO_3_)-type lithium lanthanum titanate, like (Li, La)TiO_3_ (LLTO), shows the highest bulk lithium ion conductivity of 10^−3^ S cm^−1^ at room temperature, but the high grain boundary resistance makes total conductivity about 10^−4^ S cm^−1^ [22,23,24,25]. According to lithium ion transport properties of LLTO and structurally related materials [26], the high lithium-ion-conducting phase has an A-site deficient perovskite-type structure. Lithium ion conduction occurs due to the motion of lithium ions along A-site vacancies. The ionic conductivity is highly sensitive to the lithium content. Noteworthy is that the compound is not stable in direct contact with elemental lithium and rapidly undergoes Li-insertion with consequent reduction of Ti^4+^ to Ti^3+^, leading to high electronic conductivity. Due to this reason, Ti was substituted by Ta forming a new LLTO, namely La_1/3−x_Li_3x_TaO_3_. Recently, a crystalline, lithium-stable, fast lithium-ion conductor La_1/3−x_Li_3x_TaO_3_ directly with a thin copper foil current collector has been demonstrated for a lithium-free solid-state battery [27]. The conductivity can reach 1.5 × 10^−5 ^S cm^−1^ at room temperature, which is 15 times higher than amorphous lithium phosphorus oxy-nitride (LiPON) compositions (see Figure 4).

#### 2.1.4. Sulfide Glass

**Figure 5:**
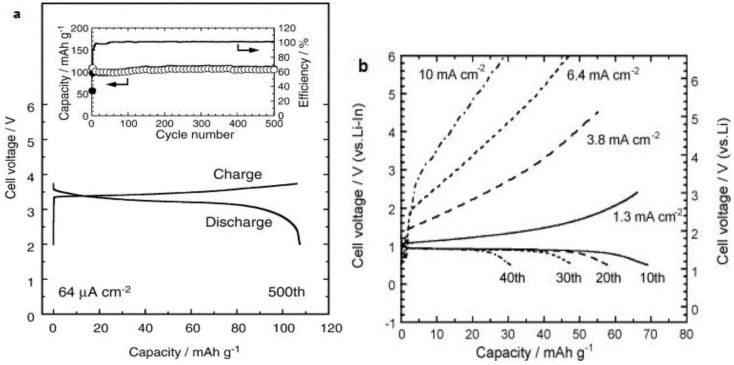
(**a**) Charge-discharge curves and cycling performance at 64μAcm^−2^ for the 500th cycle of In/LiCoO_2_ cells with 80Li_2_S-20P_2_S_5_ glass-ceramic [28]; (**b**) Charge-discharge curves of the all-solid-state Li-In/70Li_2_S-27P_2_S_5_-3P_2_O_5_/Li_4_Ti_5_O_12_ cell (discharge always at 64μAcm^−2^) [35].

Effective ways to improve the Li ion conductivity in Li_2_S-based oxysulfide glasses and sulfide glass-ceramics have been discovered: (i) the combination of sulfide and oxide anions; (ii) the replacement of an oxide matrix by a sulfide one; and (iii) the precipitation of super-ionic metastable crystals [28]. Li_2_S-P_2_S_5_ systems have shown very good Li ion conductivity, higher than 10^−3^ S cm^−1^ [29,30,31]. The exploration of some other sulfide glasses has also been demonstrated, like Li_3_PO_4_-Li_2_S-SiS_2 _glass [32], LiI-Li_2_S-Ga_2_S_3_-GeS_2_ [33], and LiI-Li_2_S-Sb_2_S_3_-P_2_S_5_ [34], *etc.* The rate performances of all-solid-state cells by the utilization of highly conductive glass-ceramic electrolytes were investigated by scientists from Japan. Although Li_2_S-P_2_S_5 _electrolyte based LiCoO_2_-In battery showed very stable capacity within 500 cycles, the charge and discharge current density is very small (Figure 5a) [28]. Li-In/Li_4_Ti_5_O_12_ cell using 70Li_2_S-27P_2_S_5_-3P_2_O_5_ solid electrolyte exhibited very poor rate performance (Figure 5b) [35], which is probably caused by disconnected ionic pathway within the electrode. 

**Figure 6:**
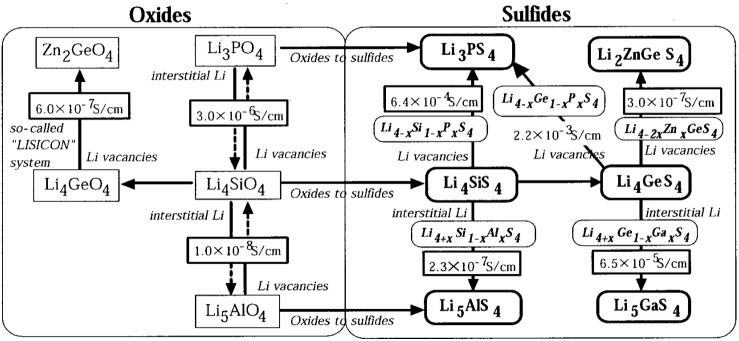
The concept of material design for the lithium superionic conductor (LISICON) system, and materials belonging to the LISICON (oxides) and the thio-LISICON (sulfides) are summarized [36].

Lithium superionic conductor (LISICON) [Li_14_Zn(GeO_4_)_4_], an important Li ion conductor, is a general name for the glass-ceramic system. Based on some criteria of material design of crystalline ionic conductors (Figure 6): (1) mobile ions should have a suitable size for conduction pathways in the lattice; (2) there should be disorder in a mobile ion sublattice; and (3) highly polarizable mobile ions and anion sublattices are preferable, a new crystalline material family, thio-LISICON, was found in the Li_2_S-GeS_2_-P_2_S_5_ system, which showed the highest conductivity of 2.2 × 10^−3 ^S cm^−1^ at 25 °C together with negligible electronic conductivity, high electrochemical stability, no reaction with lithium metal, and no phase transition up to 500 °C [36].

### 2.2. Solid Polymer Membranes

**Figure 7:**
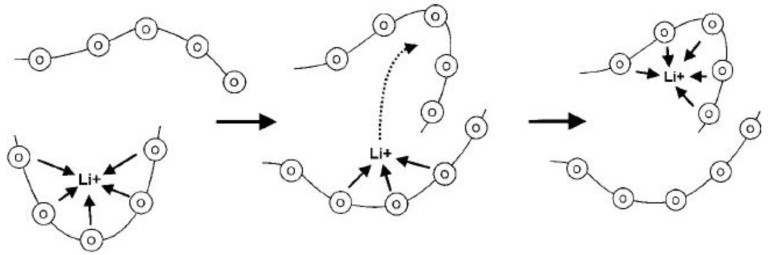
Schematic of the segmental motion assisted diffusion of Li ions in the poly(ethylene oxide) (PEO) matrix. The circles represent the ether oxygens of PEO [45].

Containing no organic liquids, such polymer electrolyte is dry and solid. Poly(ethylene oxide) (PEO) is the earliest and most extensively studied system. The ionic conduction of PEO was discovered by Fenton in 1973 [37]. Since then, a lot of work has been done to increase the conductivity of dry polymer electrolytes and reduce the cost of manufacturing of devices incorporating such electrolytes. These complexes are formed by dissolving a lithium salt, LiX, in a PEO polymer matrix to form Li ion conductor for the application of lithium rechargeable polymer batteries [38,39,40]. Definitely the lithium salt properties are critical to optimizing these materials for electrolyte applications. The large soft anions are usually preferred to improve ionic conductivity of PEO-LiX polymer electrolytes, for example, the conductivities of PEO with LiClO_4_ are within 10^−8^ to 10^−6^ S cm^−1^ [41,42], while those of PEO with Li(CF_3_SO_2_)_2_N (LTFSI) [43] and Li(C_2_F_5_SO_2_)_2_N [44] are between 10^−5^ to 10^−4^ S cm^−1^. The mechanism of Li ion transport can be described as the motion of the Li ions between complex sites assisted by the segmental motion of the PEO matrix (see Figure 7) [45]. According to this model, the good conductivity can be ascribed to Li ion transport in the amorphous region in PEO. Therefore, the Li ion conductivity can be increased in two ways: (1) reducing crystallization of PEO and (2) weakening the interaction between Li ions and PEO chains. For the former, adding plasticizer [46] or inorganic oxides [47] may achieve that purpose while employing room temperature ionic liquid [48] can affect the interaction of Li ions with polymer. 

## 3. Microporous Separators

This type of membranes usually works as the separator in liquid electrolyte batteries. The separator must physically keep anode and cathode from contacting with each other, while enable free ionic transport. Based on the morphology of the separator, there are generally two kinds of separators including microporous membranes and nonwoven films. Although separators are effective in preventing electrical shorts between anode and cathode, their presence in between the two electrodes decreases the effective conductivity of the electrolyte, raising cell impedance. This would be expected since the presence of the separator decreases the total cross sectional area of lithium ion conducting pathway, and the tortuosity of the open pores in the separator prolongs the ionic transport pathway. For this reason, one can imagine that the thinner the separator, the higher the ionic conductivity. However, it has been pointed out that there is a trade-off between the thickness of the separator and its mechanical properties. The detailed requirements for the separator in liquid lithium ion batteries are listed in Table 1 [49].

**Table 1:** Separator requirements for liquid lithium ion batteries (LIBs).

Parameter	Target	Note
**Thickness (µm)**	<25	–
**MacMullin number**	<8	The ratio of the resistance of the separator filled with electrolyte to the resistance of the electrolyte alone
**Gurley (s)**	~25/mil	The time required for air to pass through the separator
**Pore size (µm)**	<1	–
**Porosity (%)**	~40	–
**Puncture strength (g/mil)**	>300	–
**Melt integrity (°C)**	>150	–
**Chemical stability**	Long enough time	–
**Thermal stability**	<5% shrinkage	–
**Tensile strength**	<2% offset at 1,000 psi	–
**Skew (mm/m)**	<2	–

### 3.1. Microporous Membranes

The materials used for the microporous polymer membranes are semi-crystalline polyolefin materials, like polyethylene (PE), polypropylene (PP) and their blends PE-PP. The preparations of the microporous membranes can be classified into dry process and wet process. A thorough overview of these techniques has been provided previously [49,50]. In the dry process, the melted polymer is firstly extruded into uniaxially oriented tubular film. After a subsequent annealing at a temperature slightly lower than the melting point, the film was then stretched to form micropores through the orientation steps of cold stretch, hot stretch and relaxation. The wet process involves the following procedure: first, mixing polymer resins with paraffin oil, antioxidant *etc.* to form the homogeneous solution; second, extruding the solution to get the gel-like film; third, extracting the paraffin oil and additives to obtain the targeted microporous film. 

**Figure 8:**
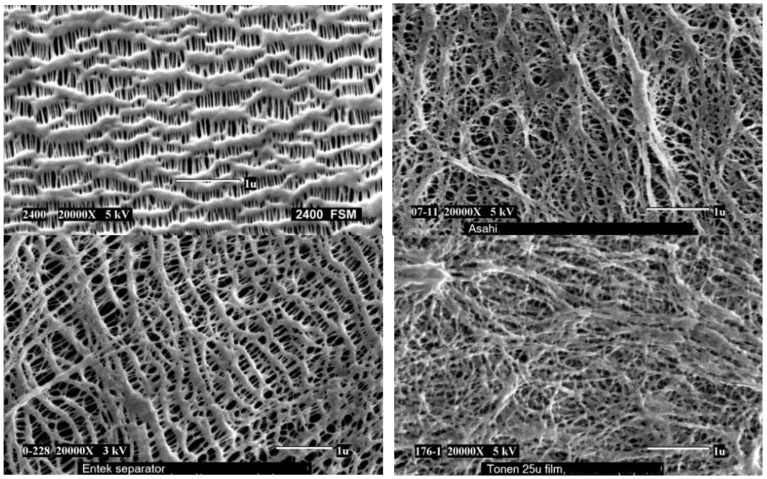
Scanningelectron micrographs (SEMs) of (**a**) Celgard separator using dry process; (**b**) Asahi separator using wet process; (**c**) Entek separator using wet process; (**d**) Tonen separator using wet process [49].

Table 2 lists some commercial separators based on microporous polyolefin membranes from 5 major manufacturers. Figure 8 shows scanning electron micrographs (SEMs) of some commercial separators [49]. The most attractive feature of the commercial separators is the fuse function, which is very important for protection from the external short-circuit and cell over-charge. Once the temperature reaches the melting point of the separator, the polymer can be flowable, and the pores will collapse forming less porous film or even a dense film, which decreases the ionic conductivity of the battery, stops the electrochemical and chemical reactions and thus protects the battery from thermal runaway. PP and PE separators have shutdown temperatures at about 165 °C and 135 °C respectively [51]. Another research topic based on commercial separator is concerned with the modification of polymer surfaces. To improve the compatibility of membranes/electrodes interfaces and electrolyte holding ability between the electrodes in the Li-ion cell, some functional groups have been attached on the polymer surfaces by electron beam irradiation and plasma [52,53].

**Table 2:** Some commercial separators.

Manufacturer	Material
Celgard LLC	PE, PP, PP/PE/PP
Asahi Kasei chemicals	PE, PP, and oxides-modified polyleffin
Entek membranes	PE, PP
SK energy	PE
Tonen	PE, PE-PP

### 3.2. Nonwoven Films

A nonwoven film is a textile product processed directly from fibers that are bonded together. The fibrous structure of nonwoven materials offers a high porosity, which is necessary for high electrolyte absorbance and low ionic resistance, and results in good charge/discharge cycles of the battery. In contrast to woven materials, the fibers in nonwovens are randomly distributed; an orientated microstructure does not occur. Compared with microporous membranes, a nonwoven film generally possesses much higher porosity and lower weight. The stochastic arrangement of the fibers is the main advantage of a nonwoven material compared with woven structures for the battery separator applications. Furthermore, it is convenient to prepare composite separators by using organic and inorganic materials simultaneously. However, the nonwoven separators have some drawbacks, such as large pore size and thicker nature. The tendency of particle penetration through the separator and the formation of dendrites during over-charging are very high in the Li-ion system. For this reason, membranes with small pores must be used. Table 3 gives the techniques of nonwoven film fabrication, related synthetic materials and film properties [50].

**Table 3:** Fabrication of nonwoven films.

Technique	Material	Film properties
Paper-making process	Polyolefin, PA, PTFE, PVDF, PVC, polyester, *etc.*	High porosity (60%–80%), large pore size (20–50 µm)
Melt-blowing method		
Electrospinning		

**Figure 9:**
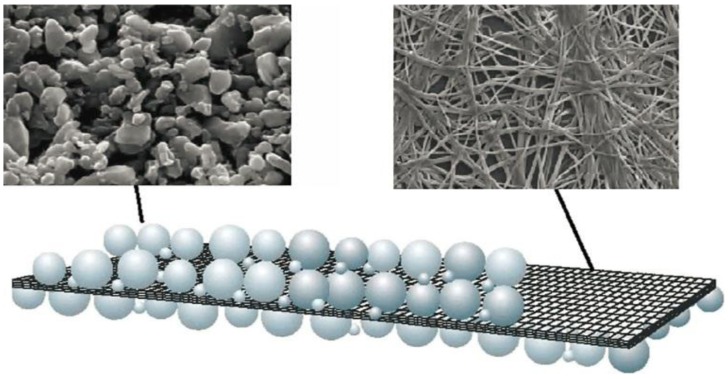
Schematic and SEMs of a Degussa composite separator [54].

Due to the disadvantages mentioned above, nonwoven films themselves are not suitable as the separator in liquid LIBs. There are two ways to employ the nonwoven films: (1) make gel polymer electrolyte as the supporting framework; (2) form composite separator by coating a layer of oxides particles on each side. Degussa commercialized a ceramic separator by coating oxides including alumina, zirconia, and silica on a thin poly(ethylene terephthalate) (PET) nonwoven film (see Figure 9) [54]. Oxide particles were obtained by hydrolyzing the precursors and suspended in an inorganic binder sol, and then the suspension was coated on a porous non-woven PET. After drying the coated PET at 200 °C a composite separator was obtained. Through this method, a separator having small pore-size, high air permeability as well as dimensional stability was developed.

## 4. Gel Polymer Electrolytes

**Figure 10:**
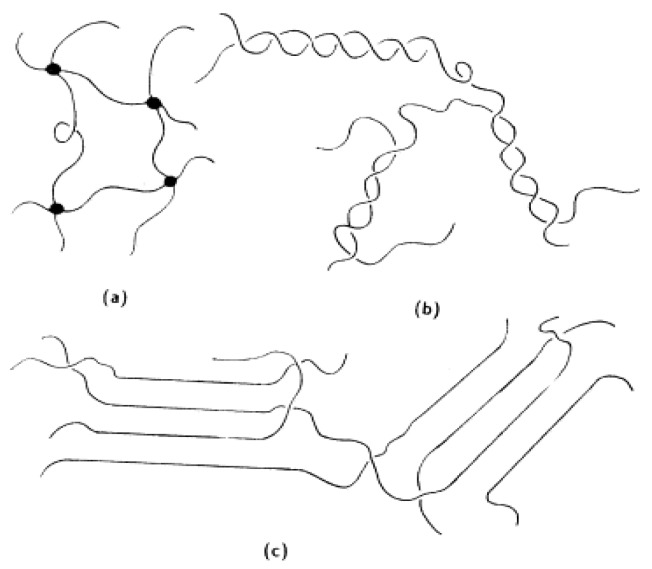
Schematic representation of (**a**) a chemical gel network with junction points; (**b**) physical gel networks having junction zones and (**c**) fringed micelles, respectively [55].

The polymer gel is formed by incorporating liquid electrolyte into the polymer matrix. The ionic conduction mechanism in polymer gels should be very similar to that in liquid electrolytes, and gels have better shape flexibility over liquids. Typically, liquid electrolyte is restrained by polymer chains within the polymer gel. Based on different structures, there are three gel networks (see Figure 10) [55]. The liquid electrolyte can also be restricted by nanofibers, as mentioned in the section of nonwoven films. 

Bellcore’s plastic Li-ion battery is a successful demonstration of the use of copolymer of vinylidene fluoride with hexafluoropropylene (PVDF-HFP) [56]. The addition of HFP introduces the amorphous domains into the polymer, which enables improved uptake of liquid electrolyte and thereby ionic conductivity. The crystalline regions provide enough mechanical integrity, and an overall plastic self-standing appearance, eliminating the need for cross-linking. A lot of work has been done to try to increase the conductivity of PVDF-HFP gels, such as changing the polymer matrix [57], adding oxide particles like Al_2_O_3_ and SiO_2_ [58], incorporating fully cyanoethylated cellulose derivative (DH-4-CN) [59], electronspinning BaTiO_3_ based PVDF-HFP [60], adding cross-linked dipoxy polyethylene glycol (DIEPEG) [61], and employing room temperature ionic liquids [62,63].

**Figure 11:**
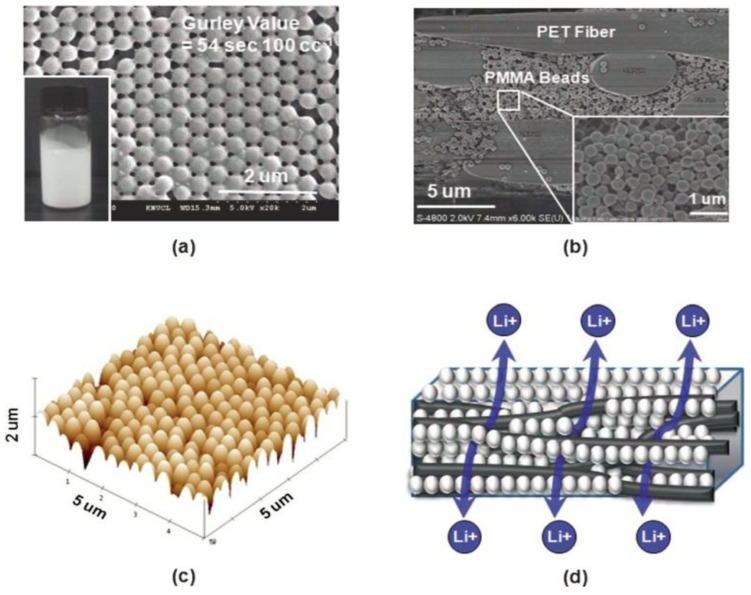
(**a**) Surface SEM of the composite nonwoven separator;the inset is a photograph of Poly(methyl methacrylate) (PMMA) nanoparticles suspension; (**b**) Cross-section SEM; (**c**) AFM photograph of the composite nonwoven separator; (**d**) Schematic illustration of nanoporous structure [69].

Poly(methyl methacrylate) (PMMA) is another important gel polymer, which has good compatibility with the liquid electrolytes, leading to good absorbing ability of the carbonate-based liquid electrolytes [64]. It is usually used to form blends [65], block copolymer [66], and layered structure to decrease the evaporation of the liquid electrolyte [67] or reduce leakage of the electrolyte [68]. A recent finding [69], in which close-packed PMMA particle arrays were introduced to a PET nonwoven separator, may inspire other research work on PMMA in LIBs. With nonwoven PET serving as a mechanical support, the well-connected interstitial voids formed between the close-packed PMMA colloidal particles in the PET nonwoven separator (see Figure 11). The highly-developed nanoporous structure and strong affinity for liquid electrolyte, the composite nonwoven separator allowed for more facile ion transport and superior electrolyte retention, which played a crucial role in improving the cell performance. 

**Figure 12:**
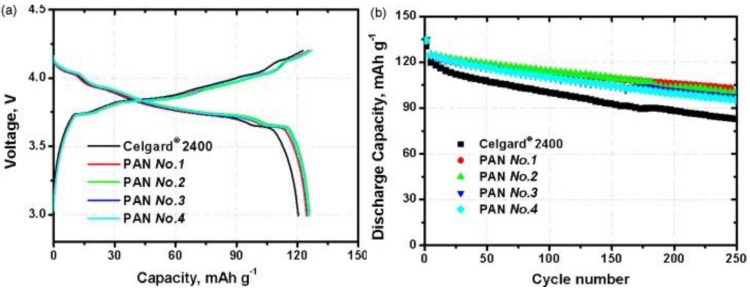
(**a**) Initial charge-discharge curves for the cells with the Celgard membrane and the Poly(acrylonitrile) (PAN) nonwoven membranes; (**b**) Discharge capacities *vs.* cycle numbers of the test cells at the 0.5C rate [78].

Poly(acrylonitrile) (PAN)-based gel electrolytes are considered of interest as separators in rechargeable LIBs. The expectation comes from the dimensional stability as well as the high conductivity at ambient (~10^−3^ S cm^−1^ at 25 °C) and subzero temperatures (~10^−4^ S cm^−1^ at −20 °C) of these electrolytes [70,71,72]. Later, the application of PAN-gel electrolyte was demonstrated in C-LiMn_2_O_4_ cell [73] and Li_4_Ti_5_O_12_-LiMn_2_O_4_ cells [74]. The drawback of polymer gel membranes is that liquid may eventually leak out from the membrane, which is deleterious both in terms of conductivity decay and, particularly, of battery reliability and safety. To solve this problem, oxide particles like Al_2_O_3_ were added in PAN-based, gel-type membranes [75]. The PAN nanofiber-based nonwoven separators for lithium-ion batteries have been developed by electrospinning technique. The cells using the PAN nonwovens showed better cycling performances than that of the Celgard due to smaller diffusion resistance of the separators [76,77,78] (see Figure 12).

## 5. Conclusions

In this study, membranes used in lithium ion batteries have been reviewed. These membranes include solid state electrolytes which contains ceramic-glass and polymer Li ion conductors, microporous separators consisting of polyolefin-based microporous separators and nonwoven films, and gel polymer electrolytes. Each type of membrane can find its position in a particular battery application, which depends on specific requirements like rigid or flexible battery design, operating temperature and desired energy and power densities. For example, microporous polyolefin separators can satisfy most common demands in the batteries for mobile electronics. Ceramic Li ion conductors may be quite suitable for micro all-solid-state batteries by employing silicon technologies. For high energy and power density batteries, *i.e.*, EV, HEV and grid energy storage LIBs, the safety requirement is a top priority, for example, present polyolefin separators cannot stand temperatures above the PP melting point (165 °C). Balancing performance, safety and cost should be the most important factors for the future research and development of LIB membranes.
